# Identifying Windows of Susceptibility by Temporal Gene Analysis

**DOI:** 10.1038/s41598-019-39318-8

**Published:** 2019-02-26

**Authors:** Kristin P. Bennett, Elisabeth M. Brown, Hannah De los Santos, Matthew Poegel, Thomas R. Kiehl, Evan W. Patton, Spencer Norris, Sally Temple, John Erickson, Deborah L. McGuinness, Nathan C. Boles

**Affiliations:** 1 Department of Mathematical Sciences, Rensselaer Polytechnic Institute, Troy, NY 12180 USA; 2 Institute for Data Applications and Exploration, Rensselaer Polytechnic Institute, Troy, NY 12180 USA; 3 Department of Computer Science, Rensselaer Polytechnic Institute, Troy, NY 12180 USA; 4 Neural Stem Cell Institute, Rensselaer, NY 12144 USA; 5 Department of Cognitive Science, Rensselaer Polytechnic Institute, Troy, NY 12180 USA

## Abstract

Increased understanding of developmental disorders of the brain has shown that genetic mutations, environmental toxins and biological insults typically act during developmental windows of susceptibility. Identifying these vulnerable periods is a necessary and vital step for safeguarding women and their fetuses against disease causing agents during pregnancy and for developing timely interventions and treatments for neurodevelopmental disorders. We analyzed developmental time-course gene expression data derived from human pluripotent stem cells, with disease association, pathway, and protein interaction databases to identify windows of disease susceptibility during development and the time periods for productive interventions. The results are displayed as interactive Susceptibility Windows Ontological Transcriptome (SWOT) Clocks illustrating disease susceptibility over developmental time. Using this method, we determine the likely windows of susceptibility for multiple neurological disorders using known disease associated genes and genes derived from RNA-sequencing studies including autism spectrum disorder, schizophrenia, and Zika virus induced microcephaly. SWOT clocks provide a valuable tool for integrating data from multiple databases in a developmental context with data generated from next-generation sequencing to help identify windows of susceptibility.

## Introduction

Development of an organism requires an intricate interplay of expansion, differentiation, and integration of numerous cellular components in order to build a typical adult. Controlling this process requires coordination of multiple signaling inputs across both time and space. Disruptions in the action or timing of these signals potentially have drastic consequences for the development of an organism. Exposure of mothers and their fetuses during gestation to a wide array of biological and chemical agents has been linked to multiple developmental disorders^1–4^. Determining the windows of susceptibility (WOS) to environmental or immune insult and the potential link to a developmental disease is a significant challenge due to the complexity of developmental systems.

Temporal analysis of expression data has focused on identifying genes that change over time and then using clustering approaches to group genes by temporal profile together. These clusters are then used in a variety of enrichment tests in combination with biological databases such as Gene Ontology (GO) to assign biological meaning to each cluster. Current bioinformatic tools such as DAVID^5^, DOSE^6^, and other enrichment tools or algorithms such as goseq^7^ can be utilized to find enrichment of ontological terms or pathways associated with a gene list. These tools in combination with disease-gene databases, such as OMIM, have been used in a wide variety of studies to try and translate gene expression changes into etiological mechanisms underlying disease. None of these approaches have attempted to identify the WOS for a disease and place the expression changes linked to a disease in a developmental context.

Currently, identifying periods of susceptibility to insult by external factors requires careful experimentation^2,8–11^. For instance, a number of studies have examined the role environmental agents play in precipitating neurodevelopmental disorders^4,12–16^. Yet, ascertaining the timing and duration of exposure needed to cause harm during human development is a difficult and costly undertaking, due to both ethical and technological constraints. However, the rise of human pluripotent stem cell (hPSC) technology and invention of protocols simulating development has provided methods to ethically and inexpensively investigate the factors contributing to developmental disorders. Proof of concept experiments testing for toxicity using hPSC developmental models have already demonstrated the validity of this system for screening possible toxins^17^. Yet, using these methods requires significant resources and time to uncover WOS and only work on a case-by-case basis.

Here, we have set out to develop a quick and cost-effective method to identify WOS. We utilize a computational method to predict WOS to injury from disrupting agents, using publicly available hPSC temporal gene expression databases along with a semantic infrastructure that combines statistical and linked data analysis^18,19^. This infrastructure links the transcriptomics data with disease-gene, pathway, gene ontology, and protein-interaction databases and then dynamically applies statistical analysis and visualizations to predict WOS for developmental diseases and to suggest potential mechanisms. The method is generally applicable to time-course expression data derived from hPSC models, which can be analyzed for WOS for a wide range of developmental disorders. As proof of concept, we utilized expression data covering a time-course of human cerebral cortex development from hPSCs^20^ and identified putative periods of vulnerability for a variety of diseases that impact neurodevelopment, including autism spectrum disorder, schizophrenia and ZIKV-virus-induced microcephaly.

## Methods

### Cortecon Dataset

For this analysis RNA-seq data from an *in vitro* human pluripotent stem cell model of cortical development, CORTECON (GEO: GSE56796), was obtained. An R session containing a matrix of normalized counts via the DESeq2 and EdgeR methods was downloaded from http://cortecon.neuralsci.org/ and used for all subsequent analysis.

### SVD analysis

Let *X* be the *m* × *n* matrix of normalized RNA expression data, where each row corresponds to a differentially expressed gene in the CORTECON dataset. Each was standardized to have mean 0 and standard deviation 1 over the time course. Standardization was done to account for different expression amplitudes between genes. Each column corresponds to a time-point. Then *x*_*ij*_ is the standardized expression level of the *i*^th^ gene in the *j*^*th*^ time-point. Computing the singular value decomposition of the data, we have *X* = *USV*^*T*^ where *U* is a *m* × *n* matrix, *S* is a *n* × *n* matrix and *V*^*T*^ is a *n* × *n* matrix. The columns of *U* are called the left singular vectors. They form an orthonormal basis of the day expression profiles. The rows of *V*^*T*^ are the right singular vectors, and form an orthonormal basis for the gene transcriptional profiles. *S* is a diagonal matrix, with the singular values in descending order down the main diagonal. The transcriptomic clock is formed by projecting the genes on the first two right singular vectors, [*x*_*i*_. *v*_1_. *v*_2_]. These vectors explain the most variance in the data. The genes are ordered in the heatmaps by their angle in the clock computed using the four-quadrant inverse tangent of their projected genes.

### Clustering analysis

The normalized counts were clustered using the Fuzzy C-Means algorithm^21^, and then each gene was assigned to its highest probability cluster. To obtain the clusters, minimize $\sum _{i=1}^{N}\,\sum _{j=1}^{C}\,{u}_{ij}^{m}{\Vert {x}_{i}-{c}_{j}\Vert }^{2}$, 1 ≤ *m* < ∞, where *x*_*i*_ is the *i*^th^ gene, *c*_*j*_ is the *j*^*th*^ cluster center and 0 ≤ *u*_*ij*_ ≤ 1 is the degree of membership of *x*_*i*_ in the cluster *j*. We selected 6 clusters using the Silhouette function in Matlab^22^. Six clusters were shown to have high silhouette values while describing the data sufficiently. Increasing the number of clusters did not improve the solution. Stability analysis used 30 trials of the algorithm made by changing the starting points based on a random seed to derive different solutions for the same C. The mean Adjusted Rand Index comparing this clustering with 30 additional trails of Fuzzy C-Means started with different random starting points was 0.996 which was close to 1 (identical partitions), indicating strong agreement between the clusters in each trial.

### Enrichment analysis

After finding the cluster membership for each gene, enrichment analysis using the CORTECON dataset as the background was provided for each disease studied. Using a contingency table (provided below), the Log Odds Ratio (LOR) was computed for each cluster and disease, as well as the p-values using Fisher’s Exact Test corresponding to the disease and cluster. If the LOR is negative, the cluster is likely to be depleted for that certain disease, with more negative numbers indicating a stronger depletion. A positive LOR indicates that the cluster is likely to be enriched for the certain disease, with more positive numbers showing a stronger enrichment. We then calculated 2-sided p-values for these LOR based on the Fisher’s Exact Test to evaluate the statistical significance of these results at the 0.05 level. From this analysis, the enrichment and depletion of the genes for each stage of development became evident. Statistically significant enrichment suggested that development during that stage was likely linked to the formation of that disease, indicating a potential window of susceptibility to the disease. For each disease p-values were adjusted across stages by the Benjamini–Hochberg procedure to control FDR.

### Semantic integration to drive visualization using a lightweight object notation for linking data

In order to ensure our pipeline and technique could easily be adapted to other developmental systems, we have taken advantage of the World Wide Web Consortium’s (W3C) recommendation for representing multidimensional data (called the Data Cube Vocabulary) which allows for easy integration of multidimensional data across organizations using a series of modular vocabularies creating semantically linked data. The cluster memberships were modeled as observations using the Data Cube Vocabulary and the associated provenance of the transformations was modeled using the W3C’s recommended language for encoding provenance (called the PROV Ontology)^23^. Using this structured representation, we generated semantic links to a number of established, curated datasets, including NLM’s Unified Medical Language System (UMLS)^24^, Ensembl^25^, Uniprot^26^, StringDB^27^, Online Mendelian Inheritance in Man (OMIM)^28^, iRefIndex^29^, DrugBank^30^, DISEASES^31^, and CORTECON^20^.

The data in these structured sources are used to construct a network of protein-protein and protein-disease interactions from a set of protein-coding genes. Such a set can be provided by specifying a list of genes or by specifying a disease, which is then semantically enhanced by identifying phenotype-genotype relations in the linked datasets. The output network structure includes the provenance of which dataset each relation originated from to provide attribution and identify areas where there is overlapping support from structured content. The output is serialized in JavaScript Object Notation with Linked Data (JSON-LD) to both publish our enhanced linkset back to the community and drive browser-based visualizations.

### SWOT clocks

Each SWOT Clock illustrates the heat map of gene transcription in CORTECON and protein-protein interactions between the genes in a specified set such as those related to a certain disease. The heat map around the outer edge of the chord diagram tracks the activity of the genes at nine intervals from the original CORTECON study: days 0, 7, 12, 19, 26, 33, 49, 63, and 77. The width of the gene is determined by the number of connections. The thickness of the chord between two genes shows their measure of connectedness, or Combined Score as determined by StringDB. Each connection has eight numerical measures associated with it: neighborhood, fusion, co-occurence, homology, co-expression, experimental, knowledge, and text-mining. The Combined Score is computed by combining the probabilities from the measures and correcting for the probability of obtaining the results by chance. The dominant cluster, or dominant stage, for each disease is defined as the cluster with positive log odds ratio with the lowest p-value using the Fisher’s Exact Test.

The SWOT Clocks are created using a Node.js application. The SWOT Clock build process is generalized to encompass arbitrary sets of genes or predefined sets pertaining to either a disease or a KEGG pathway; the only requirement is that the input exists in the database. The diagrams are created from the JSON-LD data obtained by consuming the API described above. The visualization relies on D3.js, an open source JavaScript library for manipulating HTML and SVG objects based on attached data to create powerful visualizations on the web (https://SemNExT.tw.rpi.edu/swotclock/). The chord structure in the center of the diagram uses the D3 Chord Layout to calculate and draw the arcs between the genes. The heat map around the outside of the chord diagram was created by drawing a series of arcs using the angle of each gene found by SVD. The color scale for the heat map has a domain of the mean plus or minus twice the standard deviation to avoid overcompensation for outliers. The heat map and protein-protein interaction data can be downloaded for each disease though the download function.

### Autism and Schziophrenia analysis

Gene lists for the autism^32,33^ analysis were obtained from the literature and SFARI website. In the case of the autism list from^32^ the top 200 genes (defined by reported p-values) were selected for the analysis. The gene list for schizophrenia were obtained from^34^.

### ZIKV analysis

SRA files were obtained from GEO:GSE78711, which contains Paired-end RNA sequencing data covering ZIKV infection of hPSC derived cortical progenitors^35^. Files were converted to FastQ format using the NIH sratoolkit and STAR aligner^36^ was used to map them to UCSC hg19 using gencode.v19.annotation.GTF. Differential gene expression was determined using the DEseq and edgeR packages in R, and genes with an FDR p-value < 0.05 were selected as significantly different between infected and control cells. Additional details can be found in Supplemental Experimental Procedures which contain the R code and session information. Genes significantly changing in ZIKV infected cells were identified and intersected with the microcephaly associated genes. Interactions between these genes were collected from the STRING dB along with the first degree neighbors, then the new gene list was filtered through the ZIKV and CORTECON data sets. The process was then repeated with the new list once. STRINGdb collects gene interaction data for a wide variety of organisms from a multitude of databases, for our study we used the human gene identifiers. The interaction data for the expanded microcephaly network was then downloaded and the network was analyzed using Cytoscape^37^. Communities were identified using the GLay algorithm in the ClusterMaker app^38^. Then using the AutoAnnotate app with the Word cloud-Biggest Words feature in combination with Gene Ontology categories, we identified the most associated biological processes with each community. Finally, for readability’s sake, the network was filtered by removing those nodes with a betweenness centrality less than 0.02 for display purposes only.

STAR settings and R session info and code can be found in Supplemental Experimental Procedures.

## Results

### Discovering windows of susceptibility during development

**Figure 1:**
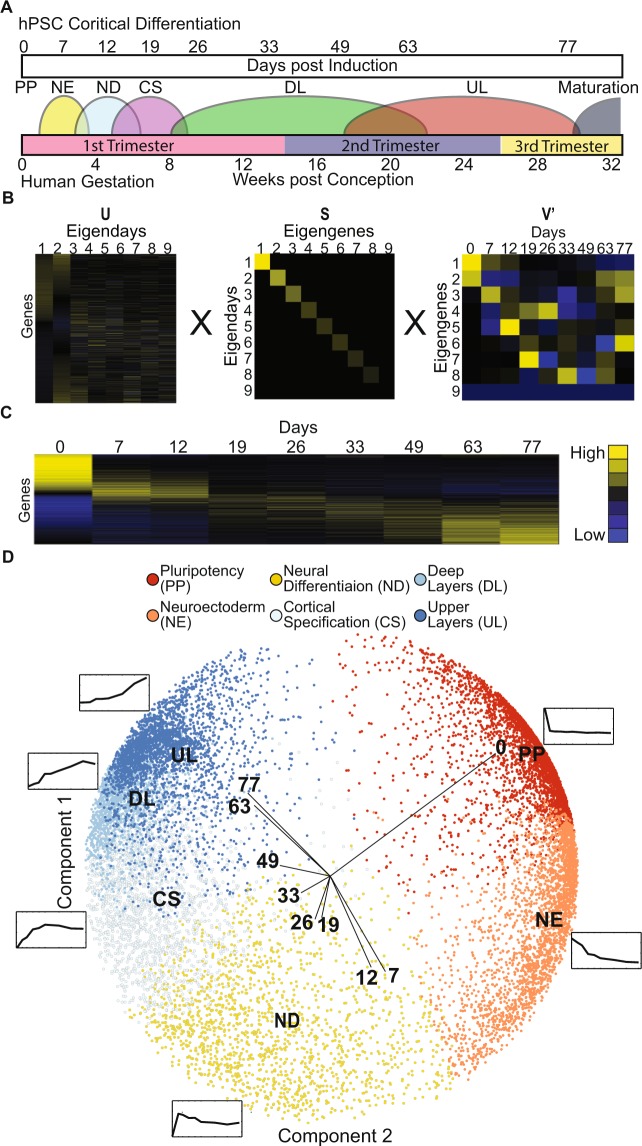
SVD reveals a developmental clock in expression data. Singular Value Decomposition (SVD) was used to analyze gene expression data derived from RNA-seq over a developmental time-course covering corticogenesis. (**A**) Diagram illustrating how each time point in the protocol relates to human development based on the marker analysis from^20^ and human cortical development^72,73^. (**B**) Heatmaps of decomposition the transcriptome by SVD into factors and virtual eigengenes that capture the variation attributed to the genes and time points (days). Matrices U and V are left and right singular vectors of the scaled expression data. The most significant eigengenes are found in the diagonals of S. (**C**) Heatmap of scaled expression data. (**D**) Corticogenesis clock formed by genes plotted in space spanned by first two left singular vectors colored by their corresponding Fuzzy C-Means clusters which match the stages of corticogenesis. The average cluster profiles from Fuzzy C-Means are shown placed at their position in the clock.

The RNA-seq expression data used in this analysis was derived from an *in vitro* model of cerebral cortical development, covering nine distinct developmental time points (days 0, 7, 12, 19, 26, 33, 49, 63, 77) identifying 14,065 significantly changing transcripts^20^. In the original study, time points were assigned to specific developmental periods parallel to human development from hPSCs to deep and upper cortical layer production (Fig. 1A) based on a marker enrichment analysis^20^. In the present analysis, expression values were the averages of at least two measurements taken for each gene at each of the 9 time points. Each gene is further normalized to have a mean of 0 and standard deviation of 1 over the time course in order to make accurate comparisons between genes with different sizes and abundances. Due to the large amount of expression changes over several time points, it is beneficial to reduce the dimensionality of the data to more clearly identify differential expression. Singular Value Decomposition (SVD) is an ideal choice for this, as it linearly reduces the data without making nonlinear assumptions about the data or needing to choose various parameters, as in kernel PCA or t-SNE, and is a well-established technique from prior studies^39,40^. We then performed a SVD analysis to precisely characterize the temporal signatures. SVD decomposes the transcriptome into factors and virtual eigengenes that capture the variation attributed to the genes and time-points (Fig. 1B). In the context of temporal expression analysis this technique has previously been utilized with cyclic biological processes occurring over the short-term^39^ but not with long term linear developmental processes such as corticogenesis. SVD uses the following equation *X* = *USV*^*T*^, where *X* is the scaled CORTECON data and the matrices *U* and *V* are its left and right singular vectors (Fig. 1). The most significant eigengenes are determined by the top singular values that are found in the diagonals of *S*. The *ith* column of *U* explains the contribution of the genes to the *ith* eigenday. The *ith* row of the transformation matrix *V*^*T*^ represents the expression of *ith* eigengene across time. SVD succinctly captures temporal dynamics of corticogenesis since 83% of the variation is explained by the first three rows of *V*^*T*^, which have a clear temporal interpretation (Fig. 1B). The first two left singular vectors are then used to order the genes. Each gene is represented by the point in the first two left singular vectors, and then the angular distance is computed from this point to the y-axis, much like time on a clock. The genes are sorted by these angular distances. The heatmap of the ordered genes clearly demonstrates this approach delineates a unique molecular signature with different waves of gene transcription during cortical development (Fig. 1C).

In order to understand more fully how gene expression changed over time, we utilized a Fuzzy C-means clustering analysis with the mean Adjusted Rand Index cluster validation technique. Fuzzy C-means’s probability assignments allow for “softer” clusters than those produced by other clustering algorithms, such as k-means. This is advantageous in situations like ours where the clusters are not easily separable. Fuzzy C-Means is an iterative clustering algorithm which allows data points to belong to more than one cluster with a certain probability. Using silhouette analysis in combination with Fuzzy C-Means clustering we determined our data contained 6 clusters with distinct molecular signatures. To visualize these signatures, each genes’s normalized expression profile is mapped in the subspace spanned by the first two left singular vectors, scaled by the singular values (columns of US) and then plotted in the subspace colored by cluster. The clusters of genes form a developmental “Clock” with the Pluripotency stage beginning at the 12 o’clock position and progressing through the stage of corticogenesis in a clockwise manner (Fig. 1D). To validate the clusters, we ran 30 trials of Fuzzy C-Means clustering and compared the resulting clusters to the clusters found in the original run of the data. To measure the overall clusters’ similarity, we calculated the Adjusted Rand Index (a similarity measure between clusters adjusted for chance) between our original partitioning of the data and each new partitioning and then averaged the results. The means of the six clusters for each of the days of the analysis shown in the six inset plots appear in a clear sequence along the hour positions of our ‘clock’ plot (Fig. 1D). These clusters have a temporal ordering directly corresponding to the five stages of corticogenesis previously identified^20^, plus one additional stage previously undescribed that includes about 1/7 of the genes. This stage was found to be enriched for processes related to Neuroectoderm using enrichment analysis by DAVID^5^. This resulted in the following stages: Pluripotency (PP), Neuroectoderm (NE), Neural Differentiation (ND), Cortical Specification (CS), Deep Layers generation (DL), and Upper Layers generation (UL) (Fig. 1D). The molecular signatures represented by this temporal transcriptomic ‘clock’ allow us to understand the temporal pattern of expression of each gene during corticogenesis with respect to these developmental stages.

The clock enables us to putatively identify the temporal roles of groups of genes in corticogenesis. Current enrichment approaches for expression data are often clustered, then checked for which diseases or terms are enriched using the entire genome or genes from a microarray^41,42^. This utilizes enrichment tools such as DAVID^5^ or GOSeq^7^. However, by taking this direction of analysis, one loses inherent structure between clusters, as is true of the relationship between stages of development and our clusters. By choosing to perform enrichment based on the subset of genes corresponding to each disease in each cluster, we will retain the link between the stages of development and disease, leading to a more accurate WOS. To achieve this the uploaded gene list is intersected with the genes in the cortecon dataset first, then the intersected gene list is used for the enrichment analysis. Thus, due to the need for each gene to be in a cluster to compute enrichment analysis, enrichment was performed using the CORTECON dataset as the background. We then identify WOS for developmental diseases by identifying groups of genes associated with developmental diseases using a literature based disease-gene association database^31^. Then, after creating an intersected gene list, we perform the gene set enrichment analysis using Fisher’s Exact test to identify which stages of development are most likely to be windows of susceptibility. To further gain insight we can also determine the windows for susceptibility for a Kegg pathway, a set of genes associated with a disease, or any set of genes. These capabilities are made available via an interactive SWOT (Susceptibility Windows Ontological Transcriptome) Clock web tool (https://semnext.tw.rpi.edu/chem-dev/).

**Figure 2:**
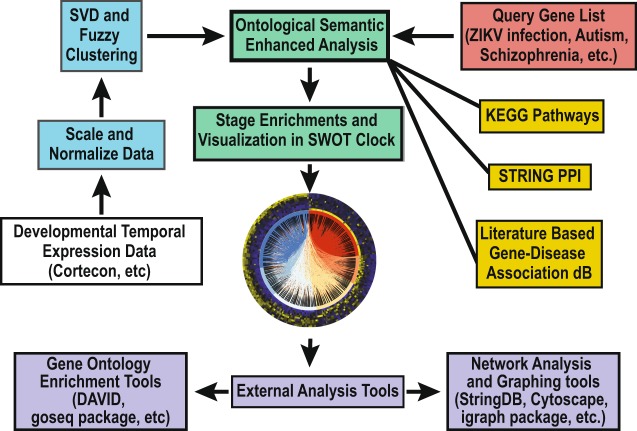
Diagram of SWOT clock tool. Basic explanation of the datasets and databases and how they are integrated to create the SWOT clock tool. Expression datasets are in red boxes, mathematical methods are in blue boxes, databases are in gold boxes, green boxes represent the integration, visualization, and enrichment phases of the tool, the white box is developmental expression data which could be substituted, and the purple boxes are for external analysis tools.

In the SWOT-Clock, the user can query the potential WOS for genes, pathways, or any groups of genes dynamically and results are presented visually and statistically in the web tool. To enable the SWOT-Clock to be highly extensible, we developed a semantic numeric exploration technology (SemNExT) approach that supports integration and linking of diverse data and ontologies from online resources closely coupled with dynamic enrichment analyses of that linked data (Fig. 2)^18^. SemNExT simultaneously links the experimental data, prior analysis results from SVD and clustering, and dynamic results from enrichment analyses with established curated datasets such as disease-gene associations, pathways, and protein-protein interactions.

The semantics powers a novel visualization called a SWOT (Susceptibility Windows Ontological Transcriptome) Clock that combines the associative visual of chord diagrams and the simplicity of a time-based heat map and leverages domain ontologies to facilitate linking between the two. First, the SWOT-Clock identifies the set of genes associated with a disease or pathway or the user can provide a customized set. Then, the SWOT-Clock determines the association of the genes with stages of corticogenesis, performs enrichment analysis of the stages as outlined above, and provides an interactive visualization of the result. The viewer is able to simultaneously analyze the connections between transcriptomes and the experimental time series data, facilitated using the SWOT Clock web tool (https://SemNExT.tw.rpi.edu/swotclock/). The chords in the center of the diagram illustrate the connections between the genes based on the STRINGdb database^27^, while the heat map depicts the RNA-Seq expression data measured over the nine time points with day 0 in the inner ring and day 77 in the outer ring. The SVD analysis orders the genes clockwise showing the wave expression moving clockwise from PP to UL (Fig. 2). The interactive web-based visualization allows the viewer to engage with the Clock by highlighting different connections to get more information about the entity or connection using the knowledge graph, and to filter connections for particular stages or genes that are of interest. Alongside the SWOT Clock, the tool also provides an enrichment analysis calculating the Log Odds Ratio and p-value for each stage in a given Clock. Using this information, we define the dominant stage as the stage with positive logs and lowest p-value. This allows the user to quickly identify stages that are significantly enriched or depleted for a disease, pathway or other transcriptome set.

Using this application in combination with a literature based disease-gene association database^31^, we performed enrichment analysis to determine whether each stage of cortical development was enriched or depleted for a neurodevelopmental disorder. This was done by computing the Log Odds Ratio (LOR) for each cluster and disease, as well as the p-values corresponding to the disease and stage. A positive LOR indicates that the cluster is likely to be enriched for genes associated with that disease, with more positive numbers showing a stronger enrichment. If the LOR is negative, the cluster is likely to be depleted for genes associated with that certain disease, with more negative numbers indicating a stronger depletion. We then calculated 2-sided p-values using the Fisher’s exact test to evaluate the statistical significance of these results. Stages with positive LOR and small p-values are identified as potential WOS to biological or environmental agents in the development of a disease (Table S1).

### Uncovering WOS for autism spectrum disorder and schizophrenia

From the SWOT clock analysis, the enrichment and depletion of disease genes for each stage of development becomes evident. Several studies have outlined similarities in the development of schizophrenia and autism (reviewed in^43–45^) indicating potential common pathways leading to each disease. Moreover, defects in the prefrontal cortex are consistently implicated in schizophrenia^46^ and autism^47^ making the CORTECON dataset ideal for querying these diseases. Autism is a neurodevelopment disorder with symptoms typically appearing in children before their third birthday and resulting in daily and lifelong struggles with the disease^48^. Autism spectrum disorder (ASD) in the vast majority (80–90%) of cases is believed to be a complex and heterogeneous disorder caused by multiple genes influenced by environmental factors^2,49,50^. The diagnosed cases of autism spectrum disorders have been on the rise in the US since the 1980’s and an impressive number of studies have looked at the contribution of environmental factors to increased autism prevalence (reviewed in^50–52^). Yet, uncovering the factors contributing to the increase in autistic patients has been hampered, as ascertaining the timing of exposure needed to cause harm during human development has been a difficult and costly undertaking.

**Figure 3:**
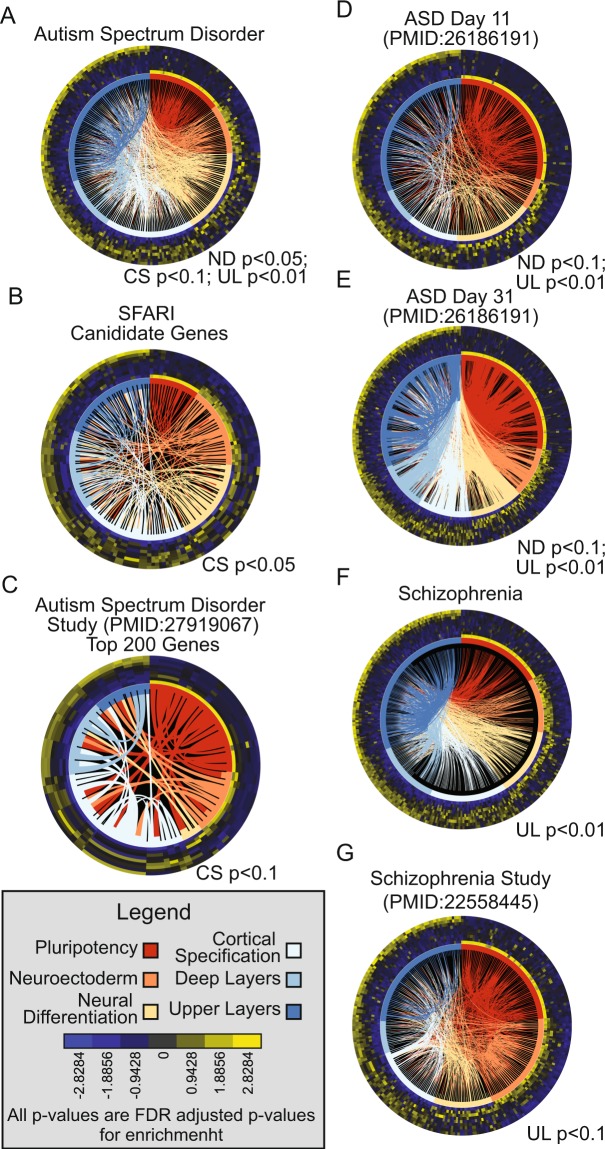
SWOT Clocks for ASD and Schizophrenia across datasets. Each SWOT clock shows the connections between genes involved in each disease while simultaneously illustrating each gene’s expression in developmental time and calculating the enriched stages and likely WOS. (**A**) SWOT clocks for genes associated with autism spectrum disorder, (**B**) SFARI candidate genes scored as 1–3, (**C**) the top 200 genes identified in a large post-mortem brain study of gene expression in ASD^32^ (**D**), and differential genes at Day 11 or Day 31 (**E**) of an organoid protocol between controls and ASD organoids^33^. (**F**) SWOT clocks for genes associated with schizophrenia (**G**) and from an expression study of schizophrenia^34^. SWOT clock for Microcephaly associated genes. (**D**) SWOT clock for genes associated with the ‘Spliceosome’ and ‘Cell Cycle’ (KEGG pathway database). Both the ‘Cell Cycle’ and ‘Spliceosome’ clock demonstrates a similar profile to the SWOT clock for Microcephaly. The initials for enriched stages in each SWOT clock are stated. All gene disease associations are from derived from the Diseases database (http://diseases.jensenlab.org/Search) unless otherwise stated. Legend: Pluripotency (PP); Neuroectoderm (NE), Neural Differentiation (ND), Cortical Specification (CS), Deep Layers (DL), Upper Layers (UL). Significant enrichment or depletion is based on a FDR adjusted p-value < 0.1.

Using the SWOT clock technology and the DISEASES database^31^, we generated an ASD SWOT clock and determined three periods during corticogenesis as likely periods of susceptibility for ASD: ND, CS and UL stages (Table 1, Fig. 3A) which are equivalent to ~4–10 (first trimester) and ~18–30 weeks post conception (mid-second to early third trimester), respectively (Fig. 1A). A previous study associated exposure to air pollution of pregnant women in the 3^rd^ trimester as a potential WOS for increasing autism risk in their fetus, which is in agreement with our prediction^53^. The SWOT Clock web tool also provides the user with the ability to upload their gene list to identify a potential WOS for their gene set of interest. This can allow researchers studying a particular disease to enter their significant genes into the tool to determine if the WOS identified using genes from the database agree with the WOS identified by their genes. This can help place their study in a neurodevelopmental context with already established disease associated genes and provide insight into the underlying etiology of the disease. To demonstrate this aspect of the SWOT clock tool, we first took advantage of the autism candidate genes identified in the SFARI database (https://www.sfari.org/resource/sfari-gene/). We took the genes scored as a 1, 2, or 3 by their gene scoring system, with 1 being highly confident and 3 being suggestive evidence, to examine with the SWOT tool. Using the SFARI genes we identified the CS stage as a period of susceptibility (Fig. 3B). We then compiled the top 200 protein coding genes by multiple test corrected p-value identified by the largest post-mortem transcriptome analysis of frontal cortex of 48 ASD and 49 control subjects^32^. Using this gene list and the SWOT clock tool, we determined a potential WOS during CS as well (Fig. 3C), which further supports the early WOS identified by ASD SWOT clock. Furthermore, the ASD study demonstrated a decrease in cortical patterning in ASD patients which agrees with CS as a critical period for the development of ASD^32^.

**Table 1:** Highlighted disease for WOS identification with Swot Clock.

Disease/Pathway		Pluripotency	Neuroectoderm	Neural Differentiation	Cortical Specification	Deep Layers	Upper Layers
ASD	log odds	−0.6105	−0.6856	**0.3684**	**0.3429**	−0.1548	**0.5814**
	fdr	0.0006	0.0008	**0.0497**	**0.0529**	0.5053	**0.0000**
SFARI ASD	log odds	−0.782	−0.1037	0.2052	**0.5521**	0.6255	0.08299
	fdr	0.0084	0.7371	0.7371	**0.018**	0.7371	0.7371
ASD Study	log odds	−0.04536	−0.2711	−0.5014	**0.8136**	0.06404	−0.2652
	fdr	1	0.8991	0.8991	**0.0858**	1	0.8991
ASD Study D11	log odds	0.1489	−1.234	**0.3796**	−0.293	−0.49	**0.6644**
	fdr	0.329	3.22E-07	**0.05565**	0.21828	0.0565	**1.56E-05**
ASD Study d31	log odds	0.03817	−0.9796	0.1687	−0.1863	−0.2289	**0.665**
	fdr	0.6569	2.83E-14	0.1626	0.1626	0.1544	**5.97E-14**
Schizophrenia	log odds	−0.5267	−0.6698	0.2424	0.1266	0.0159	**0.656**
	fdr	0.0004	0.0003	0.1689	0.4638	0.9346	**0.0000**
Schizophrenia Study	log odds	−0.09114	0.06393	0.0136	−0.01265	−0.6167	**0.3459**
	fdr	1	1	1	1	0.0498	**0.0582**
Microcephaly	log odds	−0.02455	**1.082**	−0.1372	−0.2407	−0.6943	−1.057
	fdr	0.9318	**4.05E-11**	0.7734	0.57405	0.0332	0
Spliceosome	log odds	−0.1351	**2.363**	−1.331	−1.492	−3.219	−3.748
	fdr	0.7122	**4.71E-26**	0.0139	0.0039	0	2.38E-08
ZIKV Infection	log odds	−0.1089	**0.5893**	−0.3573	−0.1085	−0.0213	−0.2774
	fdr	0.4608	**4.28E-08**	0.0484	0.57036	0.9463	0.0484
ZIKV Up	log odds	**0.3762**	**−0.9827**	−0.7971	**0.5256**	**0.4118**	−0.2389
	fdr	**0.0348**	**0.0006**	0.0184	**0.5256**	**0.4118**	0.291
ZIKV Down	log odds	−0.4443	**1.345**	−0.3621	−0.8335	−0.2096	−0.6638
	fdr	0.0054	**1.03E-25**	0.11148	0.0004	0.3156	0.0004
ZIKV + Microcephaly	log odds	−0.6459	**3.227**	−1.983	−2.144	−2.039	−2.568
	fdr	0.2716	**7.92E-13**	0.0828	0.0828	0.0828	0.0165
ZIKV + Expanded Microcephaly Network	log odds	−0.8777	**3.459**	−2.181	−2.341	−2.237	−2.765
	fdr	0.1067	**7.92E-17**	0.03408	0.03408	0.03408	0.0042

The log odds and FDR corrected p-values after Fischer exact test across time for Swot Clocks throughout the study. Bolded text represents a significant enrichment based on an FDR adjusted p-value < 0.01.

One potential confounding factor that could be affecting our analyses is the source tissue for the tested gene list could be biasing the results, i.e. a gene list from a neural progenitor could give us an earlier WOS than if the source was from adult brain. To examine the potential effect of the source tissue we took advantage of a study using brain organoids to study differentiation in idiopathic ASD hPSC^33^. In this study the authors examined two time points, one at day 11 and the other at day 31 of an organoid differentiation. The organoids at day 11 are primarily composed of progenitors (correlated primarily to 9 pcw of human brain tissue) and at day 31 a significant correlation can be seen with 16 pcw brain tissue making this dataset ideal for testing the effect of the tissue of origin on identifying a WOS. Using the differentially expressed genes between the controls and ASD at each time point we identified a WOS of ND and UL for both the day 11 (Fig. 3D) and day 31 (Fig. 3E) gene lists. This fits with two of the WOS predicated by the gene list from the DISEASES database, but doesn’t overlap with the CS WOS predicted by either the SFARI or post-mortem ASD study. This could be due to the type of organoids generated and their regional specificity, it could be an effect of using genes identified in the adult vs. a developing tissue, or it could be due to the inherent variation in ASD patients. Regardless, the WOS predicated by both the adult and organoid data fall in line with the WOS established by the ASD genes in the DISEASES database. The organoid data does demonstrate that within the same study across a broad range of developmental time; the SWOT clock technique gives the same WOS. This data proves the ability of the SWOT clock technique to consistently identify a WOS within an experiment and illustrates the flexibility of the approach to identify WOS with a variety of experimental designs.

We next sought to use the SWOT clock technique with another neurodevelopmental disease. Schizophrenia is a severe neurodevelopmental disorder characterized by defects in cognition, brain structure, and the perception of reality. The schizophrenia SWOT clock identifies a potential WOS during UL (Table 1, Fig. 3F). To further examine the WOS for schizophrenia, we took a list of genes identified by a post-mortem transcriptomics analysis of 9 schizophrenia and 9 control patients^34^, and once again identified a WOS during UL in agreement with the schizophrenia SWOT clock (Fig. 3G). The SWOT clock tool predicts a common WOS for schizophrenia and ASD during the UL stage bolster the idea of a shared mechanism. However, the SWOT clock for ASD illustrates an earlier WOS during the ND and CS stages demonstrating potential differences and alternative pathways in precipitating ASD compared to schizophrenia.

### Using SWOT clocks to identify potential disease pathogenesis

**Figure 4:**
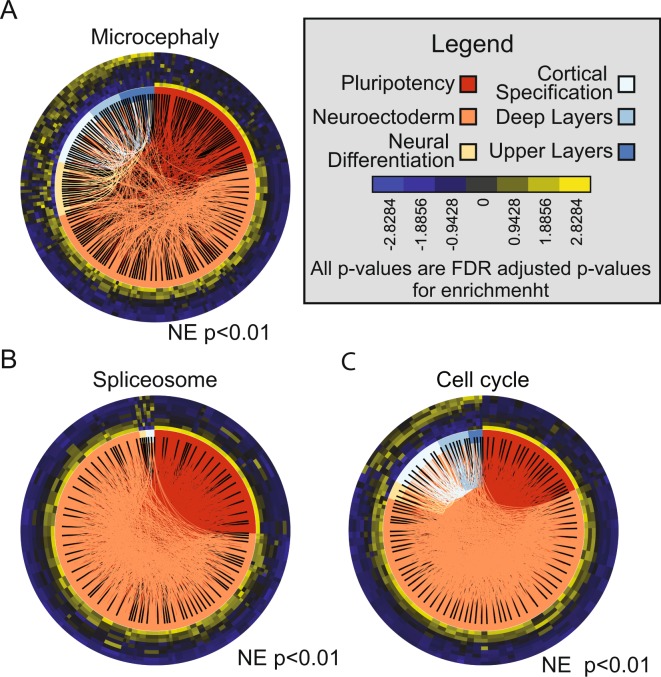
SWOT clocks related to Microcephaly. (**A**) SWOT clock for Microcephaly associated genes. (**B**) SWOT clock for genes associated with the ‘Spliceosome’ and (**C**) ‘Cell Cycle’ (KEGG pathway database). Both the ‘Cell Cycle’ and ‘Spliceosome’ clock demonstrates a similar profile to the SWOT clock for Microcephaly. The initials for enriched stages in each SWOT clock are stated. All gene disease associations are from derived from the Diseases database (http://diseases.jensenlab.org/Search) unless otherwise stated. Legend: Pluripotency (PP); Neuroectoderm (NE), Neural Differentiation (ND), Cortical Specification (CS), Deep Layers (DL), Upper Layers (UL). Significant enrichment or depletion is based on a FDR adjusted p-value < 0.1.

We examined the SWOT clock for microcephaly, a disease linked to early brain development, and found a WOS at the NE stage (Fig. 4A). In line with the predicted WOS for microcephaly, multiple studies have linked the proliferation of early neural progenitors to the pathogenesis of this disease^54^. To further investigate potential pathways underlying microcephaly, we have incorporated the KEGG pathways^55^ into the database underlying the SWOT clock tool. Using these additional analyses can indicate which signaling pathways likely contribute to disease pathogenesis by looking for overlapping enrichment between the disease WOS and the pathways. For instance, we found statistically significant enrichment for the ‘Spliceosome’ and ‘Cell Cycle’ during the NE stage overlapping with the WOS for microcephaly, further supporting a role for disruptions in splicing and cell cycle control leading to microcephaly as indicated in the literature^54,56,57^ (Fig. 4B,C). Recently, a viral infection of pregnant women has been linked to microcephaly in their offspring^4,58^.

**Figure 5:**
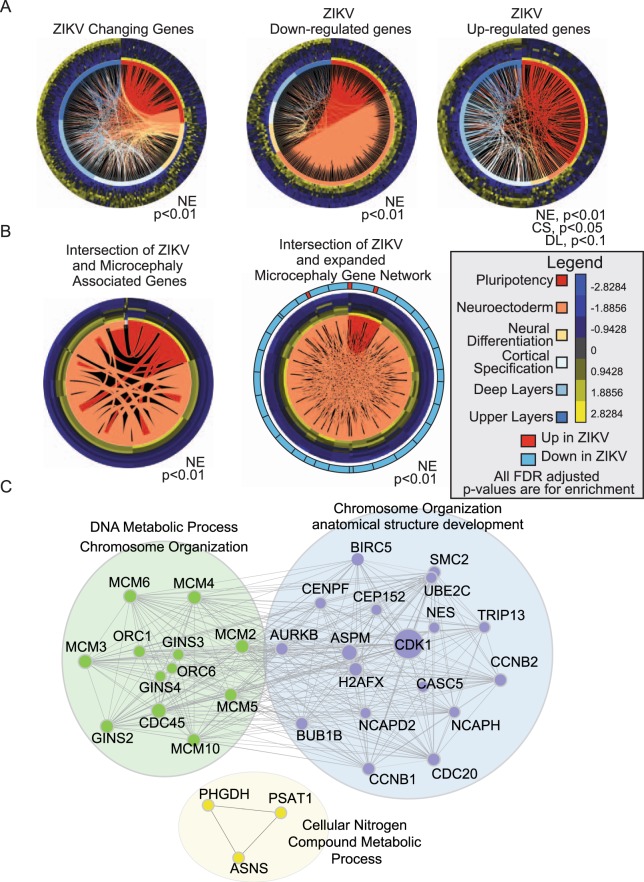
Genes down-regulated by ZIKV infection of cortical progenitors reveal a SWOT clock similar to microcephaly. Expression data for ZIKV infected cortical progenitors were re-analyzed using more stringent criteria to establish significantly changing genes and SWOT Clocks of the (**A**) all genes (left), down-regulated (middle) and up-regulated genes (right) were generated. The down-regulated SWOT Clock showed a similar profile to the Microcephaly clock. (**B**) Intersection of Microcephaly genes with the ZIKV genes filtered through the developmental time-course data (left). To examine a broader range of genes potentially contributing to the microcephaly phenotype the 1^st^ degree neighbors of the intersection SWOT clock were retrieved from STRINGdb then re-filtered through the time-course data and ZIKV data, then the process was repeated again to generate an expanded network (right). The Legend: Pluripotency (PP); Neuroectoderm (NE), Neural Differentiation (ND), Cortical Specification (CS), Deep Layers (DL), Upper Layers (UL). (**C**) Network analysis of Intersection genes shows distinct three communities contribute to the gene intersection. Significant enrichment or depletion is based on a FDR adjusted p-value < 0.1.

The recent epidemic of Zika virus (ZIKV) in Brazil became a global health crisis when patients infected with ZIKV exhibited serious neurological conditions. Multiple cases of microcephaly and Guillain-Barré syndrome associated with ZIKV infection have been reported in the Brazilian epidemic^58–62^. In response, the WHO has determined a primary objective of research efforts should be on the pathogenesis of ZIKV infection and neurological disorders^63^. Several recent studies have examined the role of ZIKV infection in the pathogenesis of microcephaly, including those using hPSC models^35^. As already demonstrated, the WOS for microcephaly is in the NE stage of corticogenesis. To further investigate the potential effects of ZIKV infection and the WOS for ZIKV related microcephaly, we retrieved recently published gene expression data obtained from human cortical progenitors derived from pluripotent stem cells infected with the ZIKV^35^ and reanalyzed using the DESeq and edgeR packages. We identified 1431 significantly changing genes, with 539 being up-regulated and 892 down-regulated in the ZIKV infected cells (Table S2). We then constructed SWOT Clocks for all significantly changing genes and both the down-regulated and up-regulated genes using the SWOT clock web tool (Table 1, Fig. 5A).

When examining both the down-regulated subset and complete set of changing genes, the WOS for ZIKV infection was identified as the Neuroectoderm stage, similar to the microcephaly SWOT Clock. Recall that the ‘cell cycle’ and ‘spliceosome’ KEGG pathways are also enriched in the Neuroectoderm stage. The down-regulated subset is also enriched for the ‘cell cycle’ KEGG pathway (Table S3), similar to the WOS genes for microcephaly. This subset, however, was not enriched for the ‘spliceosome’ KEGG pathway, illustrating that the ZIKV may not depend on altering splicing to induce microcephaly. When considering the up-regulated subset, a different picture emerges, with the SWOT Clock indicating a role for these genes in the CS and DL stages. In the up-regulated genes the ‘Protein processing in endoplasmic reticulum’ pathway is enriched and the CS stage also shows an enrichment for this pathway in agreement with the up-regulated ZIKV WOS. Overall, this data analysis establishes a very early WOS for ZIKV-induced microcephaly, which is in agreement with studies of ZIKV infection and microcephaly^64,65^.

To gain insight into how ZIKV could lead to microcephaly, we examined the intersection between known microcephaly genes changing during development and the ZIKV affected genes and found a small but significant overlap (Fig. 5B). To help address the underlying mechanisms precipitating microcephaly during ZIKV pathogenesis, we generated an expanded microcephaly network which would include genes potentially involved in microcephaly but have yet to be directly tested for involvement in or added to available databases in association with this condition. We expanded our microcephaly network by taking the 1^st^ degree neighbors from STRINGdb^27^ of the genes from the intersection, then re-filtering the new list of genes through the time-course data and ZIKV data. We then took the new list and once again took the 1^st^ degree neighbors from STRINGdb^27^ and re-filtered the expanded list through the time-course and ZIKV data to generate an expanded microcephaly network. By expanding the network in this way we only add those genes which are connected directly to ZIKV infection, microcephaly, and corticogenesis in a continuous network. We constructed a SWOT Clock of the expanded network (Fig. 5B) and found that the vast majority of genes in the network are down-regulated by ZIKV infection. Additionally, microcephaly-related genes impacted by ZIKV are most active in the earliest stages of neural development (NE stage), the foundation of building the cerebral cortex. A network analysis using Cytoscape^37^ identified three distinct communities underlying the function of intersection genes (Fig. 5C, Table S4). The two largest communities were closely related and primarily associated with “Chromosome Organization”, “DNA metabolic Processes”, and “anatomical structure development” Gene Ontology categories. This analysis reveals a subset of genes affected by ZIKV infection but not implicated previously in microcephaly as potential candidates for further study (Table S3).

## Discussion

The rise of human pluripotent stem cell technology and the continuing invention of protocols simulating development provides an opportunity to ethically and inexpensively investigate the factors contributing to developmental disorders. By integrating expression data with multiple databases and constructing a powerful visualization tool, we have developed a method to reveal potential windows of susceptibility to a variety of environmental insults leading to neurodevelopmental disorders. As a proof of concept we utilized expression data from two neurodevelopmental disease studies and a study of ZIKV infection to identify likely WOS and shed light on the etiologies of these disorders.

The SWOT clock generated from the DISEAESE database for autism identified ND, CS, and UL stages as potential WOS. Moreover, SWOT clocks generated from multiple studies and the SFARI autism candidate genes also pointed to either the ND, CS, or UL stages of development as WOS. Interestingly, the studies conducted from adult tissue and the organoid models did not identify the same WOS. In the organoid model the ND and UL stages were designated as WOS, and a recent study demonstrated that over-production of upper layer neurons led to ASD phenotypes in mice^66^. A similar increase of neurons was observed in a study of postmortem brains from children^67^, however other work suggests a loss of this phenotype with age^68^. A study in rhesus monkeys exposing mothers to an immune challenge during either the first or second trimester demonstrated behavioral defects reminiscent of schizophrenia and autism^69^. In this case the early and late exposures are occurring roughly during the same developmental time frames as the CS and UL stage, which are the WOS predicted by the ASD SWOT clocks. Each group exhibited both shared and distinct behaviors with the CS exposed group demonstrating more abnormal social behaviors in line with ASD patients and supporting the CS WOS identified by the post-mortem study and the SFARI candidate genes (Fig. 3A,B). The exposed groups also exhibited behaviors in line with the WOS identified for schizophrenia. A recent study implicated deep layer neurons in ASD pathogenesis which runs contrary to the SWOT clock prediction^70^. However, the expression data they utilized to generate the co-expression networks found an enrichment for ASD genes from 10–24 pcw. This time period has a large overlap with the UL stage (~18 pcw onward) which indicates the coexpression networks could be enriched during UL stage as well. Additionally, the design of that study chose specificity over sensitivity whereas our approach prioritizes sensitivity giving this approach an advantage in detecting more temporal defects.

The SWOT clock technique can also be used to help generate new hypothesis about the mechanism underlying disease pathology. Using the SWOT clock method we determined a WOS during the NE stage for microcephaly. Then using the KEGG pathway database we found biological pathways enriched during the same time period. Both splicing and cell cycle changes have been linked to the etiology of microcephaly previously. In the case of microcephaly induced by maternal ZIKV infection a WOS during the first trimester identified by our method was upheld by a study of 442 women in the US with likely ZIKV infection^71^. By utilizing an expanded microcephaly network, we were able to illuminate a potential role for chromosome organization in this disease and reveal multiple genes as potentially pathogenic. These results highlight the potential of our approach for ethically, inexpensively, and quickly determining potential WOS of developmental diseases. In future cases similar to the ZIKV epidemic, our approach could help identify the WOS of a developmental defect from a biological or chemical agent and provide clinicians with pertinent information for their patients.

There are limitations to our approach based on the SemNext architecture. For instance, SemNext does not directly account for biases of individual datasets and the calculated WOS depends heavily on the quality of the temporal expression data utilized as a base for the technique. For the CORTECON dataset the genes changing with time were initially isolated by EdgeR and DESeq2, but improved techniques for uncovering differentially expressed genes in a time course could yield a more accurate WOS. Our approach finds associations between developmentally changing genes, diseases, and pathways that are not likely to be the result of chance. The SWOT clock technique is designed to help in the generation of hypotheses; however, further study is required to investigate the hypotheses generated since the analysis is correlative and does not establish the underlying mechanisms. The enrichment of different stages for sets of genes does potentially reflect systematic differences for different diseases and pathways, even if individual genes are mistakenly added or missed in prior analyses. Yet, results for individual genes may not be valid due to the bias of previous studies. In the case of the CORTECON data, it is derived from a single *in-vitro* model of human development^20^, and thus more expression data from a broader set of sources would strengthen the hypotheses generated. The analysis pipeline, however, could be readily adapted to these further studies and other datasets. The method could be further improved by meta-analysis approaches and adjusting for individual uncertainties of each dataset. Future work will be focused on collating these other data sets and creating a more comprehensive tool with additional linked data and statistical analysis. The SWOT clock is available for exploration and collaboration and could be applied to a broad spectrum of developmental disorders in other organ systems as more developmental expression time course data is generated.

## Supplementary information

Supplemental Experimental Procedures

Supplementary Dataset 1

## Data Availability

All data presented in this manuscript are available from the following sources: https://semnext.tw.rpi.edu/chem-dev/; http://cortecon.neuralsci.org/; GEO: GSE56796; GEO: GSE78711; https://www.synapse.org/#!Synapse:syn4587609. Github: https://github.com/mpoegel/SemNExT-Visualizations.
