# Supplementary material for: Identifying Windows of Susceptibility by Temporal Gene Analysis
Source: Sci Rep. 2019 Feb 26;9:2740. doi: 10.1038/s41598-019-39318-8 (PMC6391370; doi:10.1038/s41598-019-39318-8)
Supplement: Supplementary file 1 — Supplemental Experimental Procedures [file 41598_2019_39318_MOESM1_ESM.docx]

**Supplemental Material For:**

**Identifying Windows of Susceptibility by Temporal Gene Analysis**

Kristin P. Bennett1,2,3, Elisabeth Brown1, Hannah De los Santos1, Matthew Poegel1, Thomas R. Kiehl^4^, Evan W. Patton3, Spencer Norris^3^, Sally Temple4, John Erickson^2^, Deborah L. McGuinness2^,3,5^, Nathan C. Boles4

**Affiliations**

^1^Department of Mathematical Sciences; Rensselaer Polytechnic Institute; Troy, NY 12180, USA

^2^Institute for Data Applications and Exploration, Rensselaer Polytechnic Institute; Troy, NY 12180, USA

^3^Department of Computer Science; Rensselaer Polytechnic Institute; Troy, NY 12180, USA

^4^Neural Stem Cell Institute; Rensselaer, NY 12144, USA

^5^Department of Cognitive Science; Rensselaer Polytechnic Institute; Troy, NY 12180, USA

***Correspondence to:** [bennek@rpi.edu](mailto:bennek@rpi.edu), [nathanboles@neuralsci.org](mailto:nathanboles@neuralsci.org)

STAR settings:

--sjdbGTFfile /home/analysis/STAR/Genomes/gencode.v19.annotation.gtf --outSAMtype BAM SortedByCoordinate --alignSJDBoverhangMin 8 --alignSJoverhangMin 16

*R Sesssion info and code:*

R version 3.2.3 (2015-12-10)

Platform: x86_64-pc-linux-gnu (64-bit)

Running under: Ubuntu 14.04.4 LTS

locale:

[1] LC_CTYPE=en_US.UTF-8 LC_NUMERIC=C

[3] LC_TIME=en_US.UTF-8 LC_COLLATE=en_US.UTF-8

[5] LC_MONETARY=en_US.UTF-8 LC_MESSAGES=en_US.UTF-8

[7] LC_PAPER=en_US.UTF-8 LC_NAME=C

[9] LC_ADDRESS=C LC_TELEPHONE=C

[11] LC_MEASUREMENT=en_US.UTF-8 LC_IDENTIFICATION=C

attached base packages:

[1] stats4 parallel stats graphics grDevices utils datasets

[8] methods base

other attached packages:

[1] org.Hs.eg.db_3.2.3 GenomicAlignments_1.6.3

[3] RColorBrewer_1.1-2 GO.db_3.2.2

[5] ggplot2_2.0.0 DESeq2_1.10.1

[7] RcppArmadillo_0.6.500.4.0 Rcpp_0.12.3

[9] SummarizedExperiment_1.0.2 DESeq_1.22.1

[11] lattice_0.20-33 locfit_1.5-9.1

[13] edgeR_3.12.0 limma_3.26.8

[15] goseq_1.22.0 RSQLite_1.0.0

[17] DBI_0.3.1 geneLenDataBase_1.6.0

[19] BiasedUrn_1.07 Rsamtools_1.22.0

[21] Biostrings_2.38.4 XVector_0.10.0

[23] rtracklayer_1.30.2 GenomicFeatures_1.22.13

[25] AnnotationDbi_1.32.3 Biobase_2.30.0

[27] GenomicRanges_1.22.4 GenomeInfoDb_1.6.3

[29] IRanges_2.4.8 S4Vectors_0.8.11

[31] BiocGenerics_0.16.1 doMC_1.3.4

[33] iterators_1.0.8 foreach_1.4.3

loaded via a namespace (and not attached):

[1] genefilter_1.52.1 splines_3.2.3 colorspace_1.2-6

[4] mgcv_1.8-11 survival_2.38-3 XML_3.98-1.3

[7] foreign_0.8-66 BiocParallel_1.4.3 lambda.r_1.1.7

[10] plyr_1.8.3 zlibbioc_1.16.0 munsell_0.4.3

[13] gtable_0.2.0 futile.logger_1.4.1 codetools_0.2-14

[16] latticeExtra_0.6-28 geneplotter_1.48.0 biomaRt_2.26.1

[19] acepack_1.3-3.3 xtable_1.8-2 scales_0.4.0

[22] Hmisc_3.17-2 annotate_1.48.0 gridExtra_2.2.1

[25] grid_3.2.3 tools_3.2.3 bitops_1.0-6

[28] RCurl_1.95-4.7 cluster_2.0.3 Formula_1.2-1

[31] futile.options_1.0.0 Matrix_1.2-3 rpart_4.1-10

[34] nnet_7.3-12 nlme_3.1-125

########create counts table########

human19<-makeTxDbFromUCSC("hg19","knownGene")

linc<-makeTxDbFromUCSC("hg19","lincRNAsTranscripts")

h19eg<-exonsBy(human19, "gene")

linceg<-exonsBy(linc, "gene")

RNA<-c(h19eg,linceg)

h19e<-exons(human19)

lince<-exons(linc)

ex.RNA<-c(h19e,lince)

SYMBOLS<-unlist(as.list(org.Hs.egSYMBOL))

x<-names(RNA[23460:45089])

names(x)<-x

SYMBOLS<-c(SYMBOLS,x)

x<-dir()

RNA.cts<-foreach(i=1:4,.combine='cbind') %do% {

bf<-BamFile(x[i],yieldSize=5000000)

open(bf)

cvg<-NULL

repeat{

yld <- readGAlignments(bf)

if (length(yld) == 0L) break

chunk.cvg<-assay(summarizeOverlaps(RNA,yld, mode="IntersectionNotEmpty", singleEnd=FALSE, fragments=TRUE, ignore.strand=F))

if (is.null(cvg)) {cvg<-chunk.cvg} else {cvg<-cvg+chunk.cvg}}

close(bf)

cvg

}

########find significantly changing genes#########

group<-c("mock","mock","zika","zika")

group<-data.frame(group)

Sue<-DESeqDataSetFromMatrix(RNA.cts,colData=group,design=~group)

colData(Sue)$times<-factor(colData(Sue)$group,levels=c("mock","zika"))

colnames(Sue)<-colnames(RNA.cts)

Sue<-estimateSizeFactors(Sue)

Sue<-estimateDispersions(Sue)

Sue<-nbinomWaldTest(Sue)

results.DEseq <-as.matrix(results(Sue))

x<-results.DEseq[which(results.DEseq[,6]<=0.05),]

DEseq.genes<-cbind(RNA.cts[rownames(x),],x)

colnames(DEseq.genes)<-c(colnames(RNA.cts),colnames(x))

group<-c("mock","mock","zika","zika")

y<-DGEList(counts= RNA.cts,group=group)

y<-calcNormFactors(y)

group<-factor(c(1,1,2,2))

design<-model.matrix(~group)

y<-estimateDisp(y,design)

fit<-glmFit(y,design)

lrt<-glmLRT(fit)

results.edgeR <-as.matrix(lrt$table)

FDR<-p.adjust(results.edgeR[,4],"fdr")

results.edgeR <-cbind(y,FDR)

x<-which(FDR<=0.05)

z<- results.edgeR [names(x),]

edgeR.genes<-cbind(RNA.cts[rownames(z),],z)

sig.genes<-intersect(rownames(edgeR.genes),rownames(DEseq.genes))

######scaled counts####

group<-c("mock","mock","zika","zika")

Sue<-newCountDataSet(RNA.cts[,5:8],group)

x<-estimateSizeFactors(Sue)

norm.cts<-t(t(RNA.cts[,5:8])/sizeFactors(x))

ave.cts<-cbind(apply(norm.cts[,1:2],1,mean),apply(norm.cts[,3:4],1,mean))

colnames(ave.cts)<-c("Mock","Zika")

######Write table of changing zika genes######

y<-ave.cts[sig.genes,]

FC<-y[,2]/y[,1]

FC<-c(FC[which(FC>=2)],FC[which(FC<=0.5)])

zika.table<-data.frame(SYMBOLS[names(FC)],norm.cts[names(FC),],ave.cts[names(FC),],results.edgeR[names(FC),5],results.DEseq[names(FC),6],FC)

colnames(zika.table)<-c("Symbols",colnames(norm.cts),colnames(ave.cts),"EdgeR FDR","DEseq FDR","Fold Change (Zika/Mock)")

write.csv(zika.table,"Table S1.csv")

#######Acquired Cortecon R data from <http://cortecon.neuralsci.org/> #####

######loaded data into R session, first added “zika.” to each R object in current session ###

#####use goseq package to find enriched KEGG pathways#####

######genes down in Zika infected cortical progenitors####

x<-intersect(rownames(sig.genes),rownames(zika.table[which(zika.table[,10]<1),]))

#########R session Finished#####
